# Supplementary material for: Nutritional Risk Screening Tools for Older Adults with COVID-19: A Systematic Review
Source: Nutrients. 2020 Sep 27;12(10):2956. doi: 10.3390/nu12102956 (PMC7599513; doi:10.3390/nu12102956)
Supplement: Supplementary file 1 [file nutrients-12-02956-s001.pdf]

Table S1: Detailed Search Strategy for Systematic Review.

Databases	Search strategy	Date	Filter applied	Results
PubMed	(“Nutrition Assessment” OR “Assessments, Nutrition” OR “Nutrition Assessments” OR “Nutritional Assessment” OR “Assessment, Nutritional” OR “Assessments, Nutritional” OR “Nutritional Assessments” OR “Assessment, Nutrition” OR “Nutrition Indexes” OR “Indexes, Nutrition” OR “Nutrition Indices” OR “Nutritional Index” OR “Index, Nutritional” OR “Indices, Nutritional” OR “Nutritional Indices” OR “Nutrition Index” OR “Index, Nutrition” OR “Indices, Nutrition” OR “Prognostic Nutritional Index” OR “PNI” OR “Index, Prognostic Nutritional” OR “Indices, Prognostic Nutritional” OR “Nutritional Index, Prognostic” OR “Nutritional Indices, Prognostic” OR “Prognostic Nutritional Indices” OR “Index, Prognostic Nutritional” OR “Indices, Prognostic Nutritional” OR “Nutritional Index, Prognostic” OR “Nutritional Indices, Prognostic” OR “Prognostic Nutritional Indices” OR “Mini Nutritional Assessment” OR “Assessment, Mini Nutritional” OR “Assessments, Mini Nutritional” OR “Mini Nutritional Assessments” OR “Nutritional Assessment, Mini” OR “Nutritional Assessments, Mini” OR “Mini Nutrition Assessment” OR “Assessment, Mini Nutrition” OR “Assessments, Mini Nutrition” OR “Mini Nutrition Assessments” OR “Nutrition Assessment, Mini” OR “Nutrition Assessments, Mini” OR “MNA” OR “subjective global assessment” OR “SGA” OR “nutritional risk index” OR “NRI” OR “malnutrition universal screening tool” OR “MUST” OR “nutritional risk screening tool 2002” OR “NRS-2002” OR “short nutritional assessment questionnaire” OR “SNAQ” OR “Geriatric Nutritional Risk Index” OR “GNRI” OR “Controlling Nutritional Status” OR “Controlling Nutritional Status score” OR “CONUT” OR “Nutrition Risk in Critically ill” OR “NUTRIC” OR “nutritional screening tools” OR “nutritional risk assessment” OR “nutritional risk screening”) AND (“COVID-19” OR “COVID19” OR “2019 novel coronavirus disease” OR “COVID-19 virus disease” OR “SARS-CoV-2” OR “2019-nCoV disease” OR “coronavirus disease 2019” OR “coronavirus disease-19” OR “COVID-19 virus infection”)	Search performed on July 3, 2020	No.	34 records retrieved
Embase	(“Nutrition Assessment” OR “Assessments, Nutrition” OR “Nutrition Assessments” OR “Nutritional Assessment” OR “Assessment, Nutritional” OR “Assessments, Nutritional” OR “Nutritional Assessments” OR “Assessment, Nutrition” OR “Nutrition Indexes” OR “Indexes, Nutrition” OR “Nutrition Indices” OR “Nutritional Index” OR “Index, Nutritional” OR “Indices, Nutritional” OR “Nutritional Indices” OR “Nutrition Index” OR “Index, Nutrition” OR “Indices, Nutrition” OR “Prognostic Nutritional Index” OR “PNI” OR “Index, Prognostic Nutritional” OR “Indices, Prognostic Nutritional” OR “Nutritional Index, Prognostic” OR “Nutritional Indices, Prognostic” OR “Prognostic Nutritional Indices” OR “Index, Prognostic Nutritional” OR “Indices, Prognostic Nutritional” OR “Nutritional Index, Prognostic” OR “Nutritional Indices, Prognostic” OR “Prognostic Nutritional Indices” OR “Mini Nutritional Assessment” OR “Assessment, Mini Nutritional” OR “Assessments, Mini Nutritional” OR “Mini Nutritional Assessments” OR	Search performed on July 3, 2020	No.	67 records retrieved.

	"Nutritional Assessment, Mini" OR "Nutritional Assessments, Mini" OR "Mini Nutrition Assessment" OR "Assessment, Mini Nutrition" OR "Assessments, Mini Nutrition" OR "Mini Nutrition Assessments" OR "Nutrition Assessment, Mini" OR "Nutrition Assessments, Mini" OR "MNA" OR "subjective global assessment" OR "SGA" OR "nutritional risk index" OR "NRI" OR "malnutrition universal screening tool" OR "nutritional risk screening tool 2002" OR "NRS-2002" OR "short nutritional assessment questionnaire" OR "SNAQ" OR "Geriatric Nutritional Risk Index" OR "GNRI" OR "Controlling Nutritional Status" OR "Controlling Nutritional Status score" OR "CONUT" OR "Nutrition Risk in Critically ill" OR "NUTRIC" OR "nutritional screening tools" OR "nutritional risk assessment" OR "nutritional risk screening") AND ("COVID-19" OR "COVID19" OR "2019 novel coronavirus disease" OR "COVID-19 virus disease" OR "SARS-CoV-2" OR "2019-nCoV disease" OR "coronavirus disease 2019" OR "coronavirus disease-19" OR "COVID-19 virus infection")			
LILACS	("Nutrition Assessment" OR "Assessments, Nutrition" OR "Nutrition Assessments" OR "Nutritional Assessment" OR "Assessment, Nutritional" OR "Assessments, Nutritional" OR "Nutritional Assessments" OR "Assessment, Nutrition" OR "Nutrition Indexes" OR "Indexes, Nutrition" OR "Nutrition Indices" OR "Nutritional Index" OR "Index, Nutritional" OR "Indices, Nutritional" OR "Nutritional Indices" OR "Nutrition Index" OR "Index, Nutrition" OR "Indices, Nutrition" OR "Prognostic Nutritional Index" OR "PNI" OR "Index, Prognostic Nutritional" OR "Indices, Prognostic Nutritional" OR "Nutritional Index, Prognostic" OR "Nutritional Indices, Prognostic" OR "Prognostic Nutritional Indices" OR "Index, Prognostic Nutritional" OR "Indices, Prognostic Nutritional" OR "Nutritional Index, Prognostic" OR "Nutritional Indices, Prognostic" OR "Prognostic Nutritional Indices" OR "Mini Nutritional Assessment" OR "Assessment, Mini Nutritional" OR "Assessments, Mini Nutritional" OR "Mini Nutritional Assessments" OR "Nutritional Assessment, Mini" OR "Nutritional Assessments, Mini" OR "Mini Nutrition Assessment" OR "Assessment, Mini Nutrition" OR "Assessments, Mini Nutrition" OR "Mini Nutrition Assessments" OR "Nutrition Assessment, Mini" OR "Nutrition Assessments, Mini" OR "MNA" OR "subjective global assessment" OR "SGA" OR "nutritional risk index" OR "NRI" OR "malnutrition universal screening tool" OR "MUST" OR "nutritional risk screening tool 2002" OR "NRS-2002" OR "short nutritional assessment questionnaire" OR "SNAQ" OR "Geriatric Nutritional Risk Index" OR "GNRI" OR "Controlling Nutritional Status" OR "Controlling Nutritional Status score" OR "CONUT" OR OR "nutritional screening tools" OR "nutritional risk assessment" OR "nutritional risk screening") AND ("COVID-19" OR "COVID19" OR "2019 novel coronavirus disease" OR "COVID-19 virus disease" OR "SARS-CoV-2" OR "2019-nCoV disease" OR "coronavirus disease 2019" OR "coronavirus disease-19" OR "COVID-19 virus infection")	Search performed on July 3, 2020	No.	No records retrieved.
